# Adaptive Self‐Sealing Suction‐Based Soft Robotic Gripper

**DOI:** 10.1002/advs.202100641

**Published:** 2021-07-03

**Authors:** Sukho Song, Dirk‐Michael Drotlef, Donghoon Son, Anastasia Koivikko, Metin Sitti

**Affiliations:** ^1^ Physical Intelligence Department Max Planck Institute for Intelligent Systems Stuttgart 70569 Germany; ^2^ Laboratory for Soft Bioelectronic Interfaces École Polytechnique Fédérale de Lausanne Geneva 1202 Switzerland; ^3^ Faculty of Medicine and Health Technology Tampere University Tampere 33720 Finland; ^4^ Institute for Biomedical Engineering ETH Zurich Zurich 8092 Switzerland; ^5^ School of Medicine and College of Engineering Koç University Istanbul 34450 Turkey; ^6^ School of Mechanical Engineering Pusan National University Busan 46241 South Korea

**Keywords:** rubber friction, self‐sealing, soft grippers, soft robotics, suction cups

## Abstract

While suction cups prevail as common gripping tools for a wide range of real‐world parts and surfaces, they often fail to seal the contact interface when engaging with irregular shapes and textured surfaces. In this work, the authors propose a suction‐based soft robotic gripper where suction is created inside a self‐sealing, highly conformable and thin flat elastic membrane contacting a given part surface. Such soft gripper can self‐adapt the size of its effective suction area with respect to the applied load. The elastomeric membrane covering edge of the soft gripper can develop an air‐tight self‐sealing with parts even smaller than the gripper diameter. Such gripper shows 4 times higher adhesion than the one without the membrane on various textured surfaces. The two major advantages, underactuated self‐adaptability and enhanced suction performance, allow the membrane‐based suction mechanism to grip various three‐dimensional (3D) geometries and delicate parts, such as egg, lime, apple, and even hydrogels without noticeable damage, which can have not been gripped with the previous adhesive microstructures‐based and active suction‐based soft grippers. The structural and material simplicity of the proposed soft gripper design can have a broad use in diverse fields, such as digital manufacturing, robotic manipulation, transfer printing, and medical gripping.

## Graphical abstract

A minimalistic suction cup design that can be used as a universal soft robotic gripper is proposed. The gripper has a thin and flat elastic membrane, which does not only create an adaptive self‐sealing with parts smaller than the gripper diameter, but can also show enhanced suction on various textured surfaces and grasping various real‐world 3D objects and delicate parts.

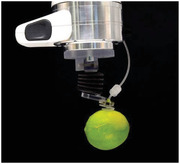

## 1 Introduction

Robust, yet reversible adhesion mechanisms on rough and irregular shapes and surfaces remain an ongoing scientific challenge for artificial smart adhesives, which can have broad applications in medical devices and adhesives,^[^ ^1^, ^2^, ^3^ ^]^ digital manufacturing,^[^ ^4^, ^5^ ^]^ robotic manipulation,^[^ ^6^, ^7^, ^8^, ^9^, ^10^, ^11^ ^]^ and transfer printing.^[^ ^12^, ^13^, ^14^, ^15^, ^16^, ^17^ ^]^ Especially, suction mechanisms have been widely used for centuries and represent one of the most common adhesion systems in our daily lives as well as robotic applications for a broad spectrum of real‐world objects.^[^ ^18^, ^19^ ^]^ Yet, suction cups often fail to secure their contact interface when engaging with irregular shapes and textured surfaces. A small opening at the interface between the gripper and the object can easily break the air‐tight sealing, leading to a suction failure. Therefore, suction cups need to be customized depending on the shape and surface texture of a target part or surface.

To overcome this fundamental limitation, various attempts have been made to achieve robust suction on rough surfaces and irregular 3D geometries.^[^ ^20^, ^21^ ^]^ In light of the efforts to improving conformability to rough surfaces, Tomokazu et al. fabricated a vacuum gripper covered with multiple microsuction cups, demonstrating gripping of irregular objects with a step or a groove in dry air, oil, and water.^[^ ^22^ ^]^ Takahashi et al. also showed improved suction of such vacuum grippers with octopus‐inspired microbumps.^[^ ^23^ ^]^ Baik et al. micromachined an elastomer surface with microsuction cups inspired by a structure of dome‐shaped protuberance inside the suckers of *octopus vulgaris*.^[^ ^24^ ^]^ For improved shape adaptation, Zhakypov et al. developed an origami‐based reconfigurable suction gripper that can actively change shape and size of the suction cup with respect to the object's geometry by actuating artificial muscle wires made of shape memory alloys.^[^ ^25^ ^]^ These previous approaches proposed various solutions for the grand challenge in developing a universal suction mechanism capable of handling a wide range of 3D irregular geometries and rough surfaces. However, the use of active components in reconfiguring the suction cups complicates the overall gripping system. Also, such active systems often involve heating to change the gripping status, requiring a relatively long time delay between picking and releasing of the objects, compared to passive systems.^[^ ^26^ ^]^ Furthermore, in addition to sophisticated microfabrication process, bioinspired adhesive surfaces with microsuction cups^[^ ^24^, ^27^ ^]^ require a high preload stress when engaging with an object, posing potential damage issues in grasping fragile objects or living tissues.

**Figure 1:**
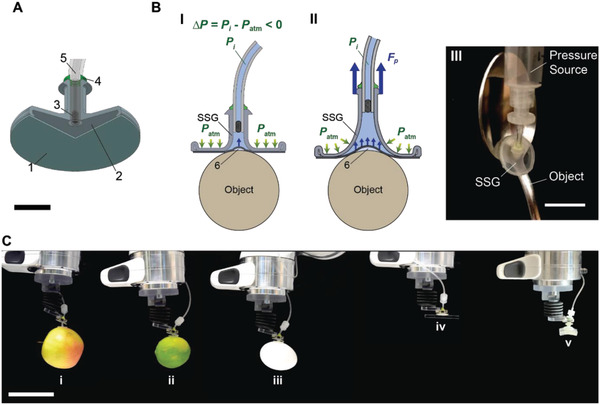
Soft suction gripper (SSG) with a self‐sealing, flat elastic membrane for pick‐and‐place of real‐world parts with various 3D shapes, sizes, textures, and materials. A) A cross‐section of the 3D assembly of the SSG. 1: flat membrane (FM), 2: suction chamber (GB), 3: filter, 4: vinylsiloxane, 5: tubing. Scale bar: 5 mm. B) A schematic image of the SSG, (I) engaging with a part smaller than the gripper diameter and (II) supporting the part's weight with expanded suction area. 6: A suction cavity between the adhering membrane and the part. (III) A photographic image of the SSG holding an irregular part with a contact area smaller than the gripper. Scale bar: 18 mm. C) The SSG holding various parts with a broad range of surface roughness, fragility, shape, and weight. i: apple (107 g), ii: lime (53 g), iii: egg (63 g), iv: rectangular bar (2 g, 5 mm in width, PMMA), and v: a block of gelatin (5 wt%, 3.5 g). Scale bar: 100 mm.

In this paper, we report a minimalistic suction‐cup‐based gripper design that can be used as a universal soft gripper. The proposed soft suction gripper (SSG) consists of a thin flat elastomeric membrane (FM, 1 in **Figure** **1**A) covering the edge of a gripper body (2 in Figure 1A). A cotton filter (3 in Figure 1A) prevents the flat membrane from being sucked into the tubing and damaged. The given SSG prototype has an overall size of 18 mm in diameter, and a 200 µm thick flat membrane made of a silicone elastomer (e.g., polydimethylsiloxane (PDMS)). Unlike previous works involving microfabrication or assembly of active components to improve suction performance, our SSG can be manufactured by simply attaching the membrane onto the gripper body. When exposed to a negative pressure differential (∆*P*), a hollow chamber inside the gripper body collapses toward the membrane, remaining only the center area exposed to ∆*P* (Figure 1B‐I). Here, a pressure differential (∆*P*) is a subtraction of the atmospheric pressure (*P*_atm_) from internal pressure (*P*_i_). ∆*P* causes the center area of the membrane to be pulled in, creating a suction cavity (6 in Figure 1B‐I). Note that the size of the suction cavity varies depending on the applied load, surface roughness, and a presence of an initial air pocket. Upon loading, the suction cavity expands toward the edge of the gripper, as deformation of the gripper body opens additional area of the FM to be exposed to ∆*P* (Figure 1B‐II). This allows the SSG to grasp various shapes of parts without having to ensure a full contact of the suction cup (Figure 1B‐III), as the suction cavity is not bound to the size of the suction cup, but to the applied load (*F*_p_). Therefore, the proposed SSG can self‐reconfigure the effective suction area without using any active components. The reconfigurability allows the SSG to achieve robust attachment to a broad range of 3D geometries with textured surfaces (Figure 1C). The SSG does not only grasp a wide range of real‐world 3D surfaces (Figure 1C,i,ii,iii; and Movie S1, Supporting Information), but can also manipulate objects smaller than its diameter (Figure 1C,iv; and Movie S2, Supporting Information). The SSG can also demonstrate pick‐and‐release of a piece of fragile gelatin without any noticeable damage (Figure 1C,v), unlike a suction cup without the FM (Figure S1, Supporting Information). The SSG can further be inflated by a positive pressure differential when releasing light parts, leveraging the high shear force of the inflating membrane at the contact interface to reduce the contact area as demonstrated in our previous work.^[^ ^28^ ^]^

Our SSG is not the first work in terms of integrating a membrane structure with a suction cup. For example, Tsukagoshi et al. have developed a ball‐shaped casting device covered with a thermoplastic elastomer that can adhere to a surface of rough wall by suction.^[^ ^29^ ^]^ Mazzolai et al. have also covered their soft suction cups on an octopus‐inspired arm with a membrane for the purpose of isolating the entire suction system from the surrounding environment and preventing hydraulic leakage for other suction cups, if one of the suckers fail to grip the surface.^[^ ^30^ ^]^ However, to the best of our knowledge, the proposed SSG is the first membrane‐based suction gripper with adaptive self‐sealing ability to change its size and the amount of suction purely based on passive deformation of the gripper body without sophisticating the gripper structure or relying on active components. This embodied physical intelligence has never been incorporated with any other membrane‐based suction grippers in previous works.

Furthermore, the proposed SSG can address a fundamental trade‐off in suction cup design between adaptability and suction force. In general, conventional suction cups with a stiff frame are designed for maximizing suction force, rather than 3D surface adaptability. The suction cup's stiff frame can create a mechanically stable suction cavity under a high negative pressure differential to achieve the theoretical maximum pull‐off force, which is equal to the amount of pressure differential multiplied by the contact area. Our SSG, on the other hand, is made of an extremely soft and stretchable silicone elastomer to maximize the surface adaptability to a wide range of 3D surfaces. However, a large deformation of the soft suction body does not allow the suction body to maintain a robust suction until it reaches to the maximum pull‐off force. In fact, a conventional suction cup architecture made of the soft elastomer could achieve only 10% of the theoretical maximum pull‐off force, as shown in Section 2.3. Therefore, a suction cup made of soft elastomer cannot achieve high suction force, and a suction cup made of a stiff material cannot conform to irregular 3D geometries. Here, we found an interesting result that a soft interfacing membrane made of the same soft elastomer can prevent the soft suction cup from sliding at the contact interface, exerting approximately 4 times higher pull‐off force. This means that our SSG can achieve both high 3D surface adaptability and suction force at the same time. This design leads to an important advancement in designing an extremely soft suction gripper for handling various 3D objects without compromising the gripping performance.

In Section 2.1, we first present basic suction characterization results of the SSG on smooth glass surfaces with different curvatures. In section 2.2, we characterize the adaptive self‐sealing ability of the SSG by measuring pressure distribution mapping at contact interface and the passive growth of the suction cavity depending on the applied load. In Section 2.3, we exhibit suction comparison between the SSG with and without the membrane in which the SSG shows significant increase in suction by the membrane. We also provide experimental observation and theoretical background on how the interfacing membrane can improve the suction. Finally, in Section 2.4, we show simulation results on the design principle of the membrane for further improved adaptability of the SSG to a higher level of surface roughness to solve diverse challenges in soft robotic gripping.

## 2 Results

### 2.1 Experimental Results on Different Surface Curvatures

**Figure 2:**
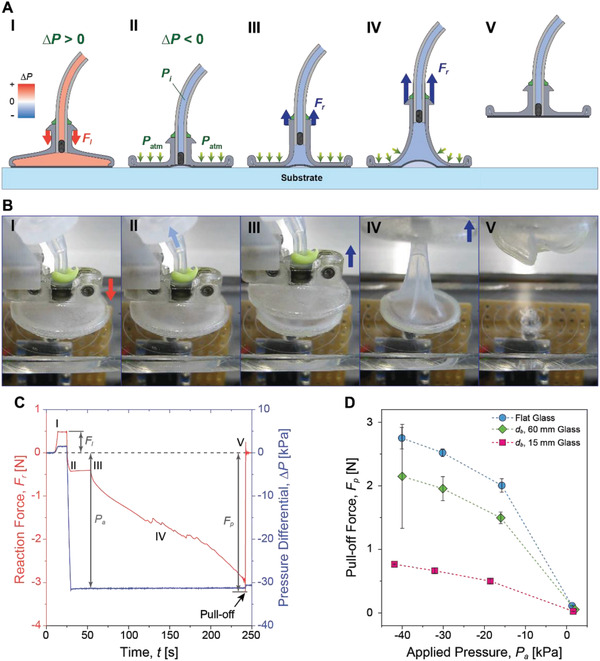
Experimental characterization of the soft suction gripper on various glass surfaces. A) A schematic procedure of a pull‐off experiment of the gripper. Preloading (I), engaging the substrate with a negative pressure differential (*∆P*) (II), initiating the retraction (III), pulling‐off the gripper (IV), and detachment (V). B) Photo images of a pull‐off experiment of the gripper on a flat glass surface, corresponding to the procedure from I to V in A. C) Profiles of reaction force (*F*_r_) and pressure differential (*∆P*) inside the suction gripper on a flat glass surface with respect to elapsed time (*t*). *I*–*V* correspond to the experimental procedure in A). D) Pull‐off forces (*F*_p_) on different radii of glass sphere surfaces depending on the applied pressures (*P*_a_). Each point indicates an average of five measurements (*n* = 5) with corresponding error bars of ±1 standard deviation (SD).

**Figure****2**; and Movie S3 (Supporting Infomation) show experimental sequence of suction measurements and basic performance testing of the SSG on smooth glass surfaces with different surface curvatures. The gripper first contacts the substrate with a preload (*F*_l_) (Figure 2A‐I,B‐I). When removing the air, a negative pressure differential (∆*P*) pulls the membrane at the center toward inside the gripper body, creating a suction cavity at the contact interface (Figure 2A‐II,B‐II). The suction cavity is a collection of residual air at the contact interface before the FM fully seals the gripper. Therefore, the size of cavity depends on the surface roughness and how much volume of air was initially entrapped at the contact interface before ∆*P* pulls the FM. When the gripper is retracted from the substrate, deformation of the gripper body opens an additional area of the membrane to be exposed to ∆*P*, resulting in passive expansion of the suction cavity (Figure 2A‐III,B‐III). Under a high pulling load, the gripper body deforms with different intrinsic curvatures evolving into a tripod‐shaped structure (Figure 2A‐IV,B‐IV).^[^ ^31^ ^]^ At the maximum pull‐off load (*F*_p_), the tripod‐shaped deformation reaches the edge of the gripper, peeling the adhering membrane off from the contact interface, and breaking the sealing on the contact interface (Figure 2A‐V,B‐V; and Movie S4, Supporting Information). The suction cavity disappears, as the pressure differential at the contact interface can no longer be maintained due to air leakage.

Figure 2C shows a representative reaction force (*F*_r_) measurement of the gripper with its corresponding ∆*P*. When approaching to a surface, the gripping system moves down until the contacting force (positive *F*_r_) reaches 0.5 N as a preload (*F*_l_). Here, we have 40 s of contact time followed by 100 µm s^−1^ of slow retraction speed to minimize the viscoelastic effects on adhesion. The ∆*P* at this moment of retraction is defined as an applied pressure (*P*_a_). During the retraction, *F*_r_ reduces due to the suction at the contact interface, and the maximum amount of *F*_r_ is defined as a pull‐off force (*F*_p_). During ≈4 min of the characterization sequence from II to IV in Figure 2C, ∆*P* changes from −31.5 to −31.2 kPa, showing a slight increase of 0.3 kPa in ∆*P* due to permeability of the silicone elastomer. Although the permeability will require a periodic recharge of a negative pressure differential inside the gripper body, it has a negligible effect on the overall performance of the gripper for temporary gripping or transferring objects as the most common usage of grippers. Figure 2D shows *F*_p_ on glass surfaces with different radii, depending on *P*_a_. The gripper exerts a higher *F*_p_ on a glass surface with a smaller curvature, as reported in previous works.^[^ ^32^ ^]^ A decrease in contact area and an increase in peeling angle attribute to the reduced *F*_p_ on a small glass sphere (Figure S2, Supporting Information). On the 15 mm in diameter glass sphere, for example, the contact area is 9 mm in diameter, while the gripper can achieve a full contact on the 60 mm in diameter glass sphere and the flat surface. Furthermore, A high peeling angle effectively reduces *F*_p_ as investigated by Kendall, K.^[^ ^33^ ^]^ A large convex surface increases the peeling angle at the edge of the gripper which results a high stress concentration, resulting in poor *F*_p_ compared to that on a flat surface (Figure S2A, Supporting Information). In case of concave surfaces, on the other hand, the soft gripper does not only remain at a full contact over the entire contract interface, but it also experiences zero peeling angle at the contact edge (Figure S2B, Supporting Information). As a result, *F*_p_ on the concave surfaces will be higher than that on the convex surfaces. The results show that *F*_p_ increases with respect to a decrease in *P*_a_ for all tested radii of curvature, demonstrating that our fully soft gripper can conform to a wide range of 3D geometries to exert a high suction force. The highest *F*_p_ among the tested surfaces is 2.8 N, or 285.7 gram‐force (gf). Considering weight of the SSG is ≈0.6 grams, the gripper can hold up to a 476 times heavier object than its body weight, which is superior to other soft suction grippers in previous works (45^[^ ^29^ ^]^ and 380^[^ ^34^ ^]^).

### 2.2 Passive Self‐Reconfiguration of the Suction Cavity at the Contact Interface

**Figure 3:**
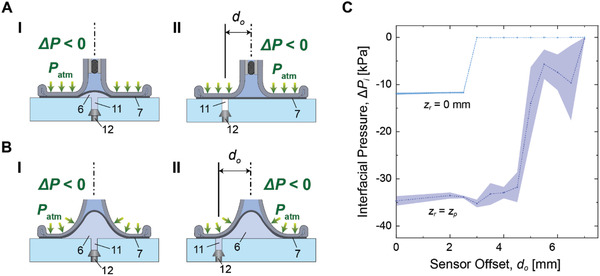
Characterization of size of the suction cavity during retraction. A) Schematic illustrations of the soft suction gripper before retraction, (I) without a sensor offset (*d*_o_), and (II) with a sensor offset (*d*_o_). 6: suction cavity, 7: membrane in contact, and 11: connecting hole, 12: pressure sensor. B) Schematic illustrations of the gripper at pull‐off (*z*_r_ = *z*_p_), (I) without a sensor offset (*d*_o_), and (II) with a sensor offset (*d*_o_). C) Profiles of interfacial pressure (Δ*P*_i_) of the gripper with respect to the gripper offsets (*d*_o_), in case when retraction (*z*_r_) is at *z*_r_ = 0 mm, and at *z*_r_ = *z*_p_. The shaded areas indicate ±1 standard deviation (SD), while the dashed lines are the arithmetic average of five measurements (*n* = 5).

**Figure****3** shows characterization of effective size of the suction cavity with respect to the applied load. As shown in Figure 3A,B, a pressure sensor (12 in Figure 3A,B) is attached on a smooth glass slide through a 500 µm diameter connecting hole (11 in Figure 3A,B) to measure the interfacial pressure (∆*P*_i_) at the contact interface. Δ*P*_i_ is measured during retraction (*z*_r_) of the gripper with different amount of sensor offset (*d*_o_). All experiments are tested with the same applied pressure, *P*_a_ = −40 kPa. Figure 3C shows profiles of Δ*P*_i_ depending on *d*_o_ from center of the gripper ranging from 0 to 7 mm. Before retraction of the gripper (*z*_r_ = 0 mm), the suction cavity exists only at the center of the gripper (Figure 3A‐I). Therefore, the pressure sensor can easily be separated from the cavity with a small amount of *d*_o_ (Figure 3A‐II). The smallest size of the suction cavity at *z*_r_ = 0 mm is ≈2.5 mm in radius, which is approximately the same size with the neck of the gripper. This means that our soft suction gripper can pick up an object whose contact area is as small as 2.5 mm in radius. Also, the amount of Δ*P*_i_ is ≈−12 kPa, far lower than *P*_a_, as the gripper body is suppressing the deformation of the membrane to create a lower pressure. When the gripper is retracted at its pull‐off point (*z*_r_ = *z*_p_), on the other hand, size of the cavity expands over the contact interface, and Δ*P*_i_ approaches to *P*_a_ (Figure 3B‐I). The pressure sensor detects Δ*P*_i_ ≈−35 kPa until *d*_o_ is ≈5 mm. When the pressure sensor is located at *d*_o_ greater than 5 mm, ∆*P*_i_ does not reach to the applied *P*_a_ = −40 kPa, since the connecting hole to the pressure sensor is reclosed rapidly by the membrane after the air inside the hole is transferred to the cavity (Figure 3B‐II). The largest size of suction cavity is estimated to be 5 mm in radius, which is 4 times bigger than the smallest cavity at *z*_r_ = 0 mm. We expect that the ratio in size between the smallest and the largest cavity can further increase by reducing the size of the neck with a smaller tubing.

### 2.3 Passive Self‐Reconfiguration of the Suction Cavity at the Contact Interface

**Figure 4:**
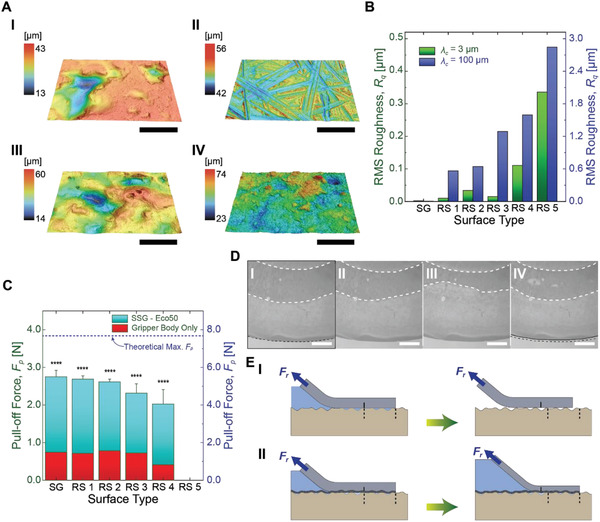
Enhanced suction performance of the soft suction gripper with the membrane on various rough surfaces. A) Topographical 3D‐scanned images of rough surfaces (RS) measured by a laser confocal microscope. (I): rough surface 1, (II): rough surface 2, (III): rough surface 3, and (IV): rough surface 4. Scale bars: 200 µm. B) Root‐mean‐square (RMS) roughness (*R*_q_) of the tested surfaces, when cut‐off wave length (*λ*_c_) is 3 µm (green) and 100 µm (blue). C) Pull‐off force (*F*_p_) comparison between the soft gripper with and without the membrane on various surfaces (rough surface (RS) 1–5) including a smooth glass (SG). The theoretical maximum of pull‐off force (*F*_p_) follows the scale of a *y*‐axis on the right. The applied pressure *P* *_a_* is −40 kPa for all measurement. Each point indicates an average of five measurements (*n* = 5) with corresponding error bars of ±1 standard deviation (SD). Statistical analysis (*t‐*test, see Table S1, Supporting Information) shows that there exists a significant difference in pull‐off force (*F*_p_) between the SSG and the gripper body without a membrane with *p*‐values < 0.0001 (marked by ****). D) Microscopic images of the soft gripper with the membrane in which the gripper body slides and relocates on the membrane. Images from I to IV are taken with 4 s of time interval. The dark area between white dashed lines shows the gripper body in contact with the membrane. Also, the black dashed and solid lines indicate positions of the contact edge before and after the gripper body slides. There is also a slight sliding at the contact edge. Scale bars: 1 mm. E) Schematic illustrations of the stick‐and‐slip of the gripper (I) without the membrane and (II) with the membrane. Blue arrows indicate tensile forces acting on the gripper body, while the dashed and solid lines show sliding of the gripper body relative to the contact interface. The shaded area in blue shows a negative pressure differential inside the gripper body.

**Figure****4** shows the experimental evidence for improved suction of the soft gripper due to the flat elastic membrane. Figure 4A shows the 3D topology of polymeric replicas of various real‐world surfaces prepared as shown in Figure S3 (Supporting Information) (see the Experimental Section for details). As shown in Figure 4A‐I, A‐III, rough surface (RS) 1 and 3 are relatively smooth in the microscale, while showing wavy surface features up to 46 µm in the maximum height difference (Figure 4A‐III). Meanwhile, rough surface 2 shows relatively flat and microscopic roughness with only 14 µm in the maximum height difference (Figure 4A‐II). Figure 4A‐IV shows rough surface 4 has both micro and macroscopic roughness up to 51 µm of the maximum height difference. Figure 4B shows root‐mean‐square (RMS) roughness (*R*_q_) of all surface samples, depending on cut‐off wavelength (*λ*_c_). While *R*_q_ with a short *λ*_c_ (i.e., *λ*_c_ = 3 µm) represents the surface roughness in microscale, *R*_q_ with a long *λ*_c_ (i.e., *λ*_c_ = 100 µm) shows more macroscopic roughness. In Figure 4B, SG stands for smooth glass, while rough surface 5 is a replica of a 2000 grit sand paper. Similar to our observation in Figure 4A, Figure 4B shows that rough surface 2 has a higher *R*_q_ in microscale, compared to rough surfaces 1 and 3. In case of the rough surface 4, although having a similar *R*_q_ to the rough surface 3 at the macroscale, the surface shows 7.4 times higher *R*_q_ at the microscale. Among all tested surfaces, rough surface 5 shows the highest *R*_q_ at both micro‐ and macroscales, 3 times higher *R*_q_ at the microscale and 1.8 times higher *R*_q_ at the macroscale than those of rough surface 4, respectively.

Figure 4C shows that the pull‐off force (*F*_p_) of the soft suction gripper with the membrane on SG and rough surfaces 1–4 has 3.8 times higher suction compared to the gripper without the membrane on average. Note that both grippers with and without the membrane readily fail on the rough surface 5 due to the high roughness. The both grippers with and without the membrane are entirely made of the identical silicone elastomer (Ecoflex 00‐50, Smooth‐On Inc.) as detailed in the Experimental Methods; and Figure S6 (Supporting Information). Under a high pulling load as shown in Figure 4D; and Movie S5 (Supporting Information), the gripper body slides on top of the contact interface while relocating its body shape to release the accumulated stress. During its relocation, random stick‐and‐slip occurs at the contact edge (Figure 4D‐IV). In case of the gripper without the membrane, this stick‐and‐slip can easily break the vacuum sealing at the contact interface (Figure 4E‐I). In case of the gripper with the membrane, on the other hand, interfacial suction brings the membrane to intimate contact with the surface and exerts friction high enough to resist the relocation of the gripper body. Therefore, the stick‐and‐slip of the gripper body occurs on top of the membrane, not on the contact interface (Figure 4E‐II). This means that the stick‐and‐slip of the gripper body does not break the sealing at the contact interface, ensuring robust attachment under a high pulling load. Considering the maximum theoretical pull‐off force (*F*_p_|_max_) can be estimated by *P*_a_ *πd* ^2^/4 (*P*_a_: applied pressure, *d*: gripper diameter), the gripper with the membrane could reach up to 36% of *F*_p_|_max_ = 7.7 N (*P*_a_ = −40 kPa, *d* = 18 mm), whereas the one without the membrane ended up achieving only 10% of *F*_p_|_max_. To the best of our knowledge, there does not exist a proper model to explain the frictional sliding of the rubber on another rubber‐like surfaces similar to our case. However, stick‐slip of rubber on rigid substrates has been studied in previous works, known as Schallamach waves.^[^ ^35^, ^36^, ^37^ ^]^

According to Viswanathan et al., interfacial shear strain *ε* of periodically sticking‐and‐slipping rubber shows the relation^[^ ^38^ ^]^ of

(1) $$\varepsilon ∼\frac{\Delta \gamma \Delta A}{2ah_{o}L_{c}G}\left(n\Delta t\right)$$

where *n* is wave generation frequency, ∆*t* is a time interval, 2*a* and *L*_c_ are width and length of the contact interface, respectively, *h*_o_ is thickness, *G* is shear modulus, ∆*γ* is the difference in surface energies between attachment and detachment, and ∆*A* is the area of sliding. Assuming the rest of the variables are constant, the interfacial shear strain *ε* will decrease with thicker *h*_o_ and higher *G*. This explains why the gripper without the membrane cannot reach to the theoretical maximum pull‐off force unlike typical suction cups. The typical suction cups are mostly made of much hard rubbers, such as nitrile rubbers whose Young's moduli are ≈50 times higher than that of the gripper body. Therefore, those standard suction cups do not slip at the contact interface during the pull‐off. On the other hand, our gripper body is made of extremely soft elastomers with a Young's modulus less than 100 kPa to maximize its adaptability on rough surfaces. The gripper body can easily reach up to the critical *ε* for slipping during pull‐off unlike the conventional suction cups. Therefore, ideal materials for the gripper body will be elastomer composites combining soft elastomers with nonstretchable, fabric‐like layer to suppress the sliding.

**Figure 5:**
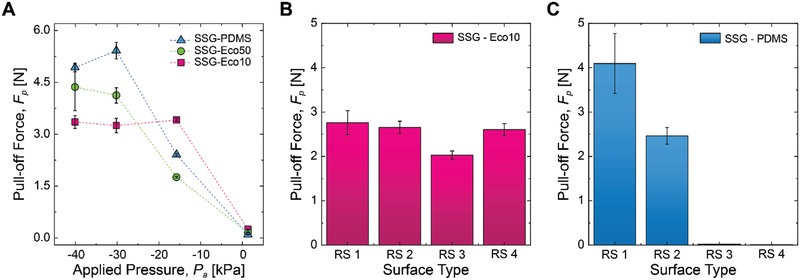
Trade‐off between maximal suction force and surface conformation/adaptation capability. A) Pull‐off force (*F*_p_) of the soft gripper on a smooth glass surface with respect to the applied pressure (*P*_a_), depending on the elastomeric material type of the flat membrane. PDMS: polydimethylsiloxane, Eco50: Ecoflex 00‐50, and Eco10: Ecoflex 00‐10. B) Pull‐off force (*F*_p_) of the SSG‐Eco10 on different rough surfaces from RS 1 to RS 4. *P*_a_ is −30.2 kPa for all measurements. C) Pull‐off force (*F*_p_) of the SSG‐PDMS on different rough surfaces from RS 1 to RS 4. *P*_a_ is −30.2 kPa for all measurements. Error bars indicate ±1 standard deviation (SD) of five measurements (*n* = 5).

**Figure****5** shows an important trade‐off between pull‐off force (*F*_p_) and surface conformation capability, depending on the softness of its flat membrane. Three SSGs are fabricated with membranes made out of Ecoflex 00‐10, Ecoflex 00‐50, and Sylgard 184 (PDMS). Young's modulus (*Y*) and elongation at break (*ε*_max_) of these elastomers are reported as follows; Ecoflex 00‐10: *Y* = 50 kPa, *ε*_max_ = 573%, Ecoflex 00‐50: *Y* = 100 kPa, *ε*_max_ = 860%, and PDMS: *Y* = 2.4 MPa, *ε*_max_ = 135%, respectively.^[^ ^39^ ^]^ Figure 5A shows that the SSG with a stiffer, and less stretchable membrane generally provides a higher *F*_p_ on a smooth glass surface at a lower *P*_a_. For example, the SSG with a PDMS membrane (SSG‐PDMS) could exert up to *F*_p_ = 5.4 N at *P*_a_ = − 30.2 kPa, while the SSG with an Ecoflex 00‐10 membrane (SSG‐Eco10) could reach up to *F*_p_ of 3.4 N that is 63% of the maximum *F*_p_ of the SSG‐PDMS at a higher *P*_a_ of −15.7 kPa. Although the SSG with a softer membrane could not achieve the *F*_p_ as high as that of the SSG with a stiffer membrane, Figure 5B,C show a clear benefit of using the softer membrane to conform to rough surfaces. Figure 5B shows the SSG‐Eco10 could adhere to all of the tested rough surfaces from RS 1 to RS 4 with an average *F*_p_ = 2.5 N, while the SSG‐PDMS failed to cling onto surfaces with high roughness (RS 3 and RS 4), as shown in Figure 5C. The above results remind a similar trend to the case of the GB based on Equation (1); a stiff and nondeformable polymer can be more robust against stick‐and‐slip at the contact interface but will sacrifice the conformability. Therefore, same as the GB, a heterogenous composite membrane that is made out of a nonstretchable material (i.e., fabrics) covered with a soft elastomer may achieve the above favorable properties to exert improved suction forces on a wide range of real‐world surfaces with high roughness.

### 2.4 Numerical Simulations for Guiding the Design of the Soft Suction Gripper

**Figure 6:**
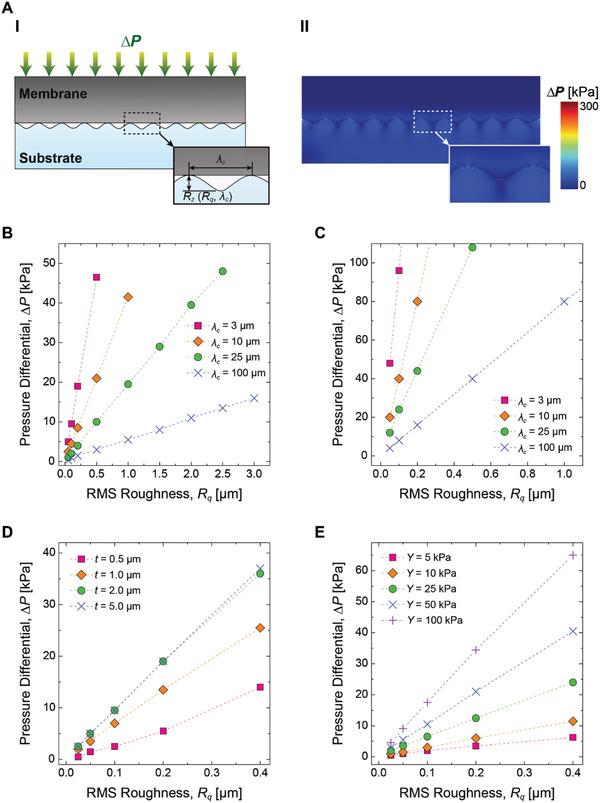
Membrane surface roughness conformability analysis based on the design parameters via finite element analysis simulations. A) Schematic of a simulation sample (I) and the result (II). The membrane is conformed to the rough surface geometry of the substrate by the applied pressure differential, Δ*P*. B,C) Membrane conformability by the cut‐off wavelength, *λ*_c_, (or the dominant roughness feature size of the surface) with B) Ecoflex 00‐30 and C) PDMS. D) Membrane conformability by the membrane thickness, *t*, in case of Ecoflex 00‐30 of 3 µm *λ*_c_ (the case of PDMS is shown in shown in Figure S9, Supporting Information). The result of membrane thicker than 5 µm is the same as for one of 5 µm, which suggests that the effect of the membrane thickness on the conformability saturates around 5 µm. E) Membrane conformability by Young's modulus, *Y*, of simulated linear materials. A softer material conforms better than a hard material given the same Δ*P* and *λ*_c_.

**Figure****6** shows effects of various design parameters of the FM, depending on surface roughness and pressure differential (Δ*P*). Figure 6A‐I depicts the geometrical parameters including the membrane thickness (*t*), roughness parameters (cut‐off wavelength (*λ*_c_) and amplitude of the wave (*R*_z_)), and applied Δ*P*. The rough surface is represented by sinewaves in 2D, assuming that the wavelength (here coincide with *λ*_c_) of the sinewave determines the dominant feature size of the rough surface. Note that *R*_z_ can be estimated as a function of RMS roughness (*R*_q_) and *λ*_c_. Given those parameters, numerical simulation calculates the minimum Δ*P* required to seal the gap between a flat membrane and the sinusoidal‐shaped substrate as shown in Figure 6A‐II.

According to experimental results in Figure 5B,C, the critical *R*_q_ on which the SSG‐Eco10 fails to adhere is estimated as *R*_q_ = 0.34 µm at *λ*_c_ = 3 µm, or *R*_q_ = 2.8 µm at *λ*_c_ = 100 µm, while the SSG‐PDMS will readily fail on a rough surface with *R*_q_ = 0.03 µm at *λ*_c_ = 3 µm, or *R*_q_ = 1.3 µm at *λ*_c_ = 100 µm, respectively. As a prerequisite for an intimate contact, the flat membrane needs to seal both micro‐ and macrosized roughness with the applied Δ*P*, which can be described as *R*_q_ at *λ*_c_ = 3 µm, and *R*_q_ at *λ*_c_ = 100 µm, respectively.

Figure 6B,C show the calculated Δ*P* of soft and rigid membranes for the membranes to fully conform to the sinusoidal‐shaped roughness, depending on *λ*_c_ and *R*_q_. The results in both graphs show that *R*_q_ at a smaller *λ*_c_ requires a higher Δ*P* than *R*_q_ at a greater *λ*_c_, which indicate that microsized roughness is more difficult to be adapted than macrosized roughness. Furthermore, curves in Figure 6B have smaller slopes than those of curves in Figure 6C, as the soft membrane possesses better conformability than the rigid membrane. At Δ*P* = 30 kPa, which is a pressure differential applied in the experiments (Figure 5), Figure 6B shows that the soft membrane made out of Ecoflex elastomer can seal the all macrosized roughness calculated up to *R*_q_ = 3.0 µm with the given Δ*P*, while still experiencing difficulties in conforming to microsized roughness greater than *R*_q_ = 0.32 µm (i.e., RS 5), which well matches with our experimental observation (Figure 5B). In case of the membrane made out of PDMS (Figure 6C), on the other hand, the numerical results predict the rigid membrane will fail on a surface with *R*_q_ greater than *R*_q_ = 0.04 µm at *λ*_c_ = 3 µm, and *R*_q_ = 0.38 µm at *λ*_c_ = 100 µm, implying that the membrane fails on RS 3, RS 4, and RS 5 in Figure 5C due to its high macroscale roughness, which is up to *R*_q_ = 1.3 µm at *λ*_c_ = 100 µm (Figure 4B).

Numerical analyses in Figure 6D,E suggest how to further improve the adaptability of a flat membrane with various design parameters, such as Young's modulus (*Y*) and membrane thickness (*t*). Figure 6D shows predictions on the minimum *R*_q_ of the Ecoflex membrane to seal the roughness in microscale ranging from *R*_q_ = 0.0 µm to *R*_q_ = 0.4 µm at *λ*_c_ = 3 µm, depending on various *t*. The results suggest that thickness of the soft membrane needs to be significantly lowered down to *t* = 1.0 µm to achieve adaptability high enough to adhere on to the roughness of RS 5 with Δ*P* = 30 kPa. Furthermore, the effect of lowering thickness on improving 3D surface adaptability will be saturated at *t* = 5.0 µm. For a membrane with thickness above *t* = 5.0 µm, elasticity of the membrane will play a dominant role, rather than the thickness. This result suggests that thickness higher above *t* = 5.0 µm will have the similar low adaptability to *t* = 200 µm, as shown in Figure 5B. Such an extremely thin, flat membrane over a large surface area is not realistic, as its structural integrity can easily be compromised under a high loading condition, failing to withstand repetitive gripping tasks. Therefore, a structural reinforcement must be combined with such an ultra thin, membranous structure in order to exploit the high surface adaptability with sufficient mechanical stability. More realistic approach can be found in Figure 6E, showing the estimated Δ*P* of a 200 µm thick membrane to seal the same microsized roughness, depending on various *Y*. The result shows a clear negative correlation between *Y* and *R*_q_ with a given Δ*P*. Reducing *Y* by a half (25 kPa) from the current version of the SSG with Ecoflex 00‐30, our numerical estimation predicts that the flat membrane can grip all the rough surfaces presented in our experiments, although softness of membrane might limit the maximum pull‐off forces as shown in Figure 5A.

Overall, the numerical simulation results shown in Figure 6 provide a design guideline that surface conformation/adaptability of the SSG can be further improved by reducing thickness or stiffness of the membrane, as long as the membrane can endure the applied loading conditions. Also, the competing relationships among adaptability, mechanical integrity, and pull‐off force leave the fact that there would be optimal design parameters for a given target range of surface roughness that the SSG needs to grip. Furthermore, these results support our discussion in Figure 5 that a composite membrane consists of an extremely soft material with a nonstretchable fabric reinforcement can indeed achieve both high pull‐off force and better conformability simultaneously, which is a future work.

## 3 Discussions and Conclusion

Experimental and numerical results presented in this work spark a discussion about how to design a soft suction cup as a universal soft robotic gripper for real‐world objects. Suction cups are everywhere; its versatility and usability in our daily lives can easily be found in various locations for different purposes, ranging from holding a car navigation system on a wind shield, attaching a hook on a mirror in the bathroom, to transferring a thin and wide, fragile glass panel in a liquid crystal display (LCD) factory. Those conventional suction cups that consist of rigid plastic parts and hard rubbers with MPa range of Young's Modulus are designed to hold high suction force on smooth surfaces with a small curvature. For robotic grasping, however, we can clearly agree on the need of soft and conformable materials as building blocks of the suction cup to achieve higher adaptability on irregular geometries and rough surfaces. Unfortunately, the high adaptability comes with a cost; Figure 4C showed that a suction cup made of a highly deformable silicone without a membrane could not keep an applied negative pressure differential inside the gripper body under a high pulling load, reaching only up to 10% of its theoretical maximum pull‐off force.

Rather complying with such a trade‐off, here we propose a simple, but versatile SSG that can grasp a wide range of 3D geometries and textured surfaces without compromising its gripping performance. The proposed SSG leveraged deformation of its gripper body in a way that it can self‐reconfigure its effective suction area ranging from 2.5 to 5.0 mm in radius with respect to an applied load. This allowed the SSG to achieve robust attachment without the need of the entire suction area to be sealed, while commercial suction cups require customization for each part's shape and size. This passive reconfigurability could also simplify the manipulation tasks for a wide variety of real‐world objects without using sensor input and sophisticated closed‐loop control. We also show that the SSG has up to 3.8 times higher suction on various textured surfaces than that of the suction cup without the membrane. We observed that the SSG allows the gripper body to freely slide on top of the membrane while keeping the contact interface fully sealed. Due to high surface conformation of the soft membrane and enhanced suction performance, the proposed SSG could grip a broad range of textured surfaces with roughness up to 51 µm of the maximum height difference. This is a significant improvement in terms of versatility from our previous work,^[^ ^40^ ^]^ which could only pick up glass‐like, smooth surfaces. Furthermore, the SSG could demonstrate its ability to handle delicate objects, such as a hydrogel piece, without causing any noticeable damage, unlike a suction cup without membrane.

Our work proposes a design approach to develop a universal suction cup that does not have to be fully sealed with adaptive self‐sealing capability, while achieving both superior 3D surface adaptability and high suction force at the same time. Most of the existing soft grippers rely on mechanical interlocking between the gripper and an object.^[^ ^41^ ^]^ This means that geometrical relationship between the gripper and the object plays a critical role in gripping performance. For example, a soft gripper cannot achieve a secure mechanical interlocking with an object bigger than the gripper size. Therefore, soft grippers based on controllable adhesion, such as suction, have significant benefits in picking‐and‐releasing of various objects, even larger than the gripper size.^[^ ^42^ ^]^ However, as discussed in the introduction, conventional suction cups have a difficulty in handling objects smaller than the suction cup size, as their suction area needs to be fully sealed. The proposed SSG, on the other hand, can grip objects both smaller and larger than the size of the gripper. Therefore, our self‐adaptable suction gripper design has a distinctive advantage over both soft grippers replying on mechanical interlocking and the conventional suction cups. Also, same as gecko‐inspired microfiber adhesives,^[^ ^43^, ^44^ ^]^ the proposed SSG made out of nontacky silicone elastomers is washable and reusable, suitable for repetitive gripping tasks. Furthermore, despite the current prototype is not optimized for achieving high durability, the SSG can withstand over 160 times of repetitive gripping tests with reliable suction force, since the elastic gripper body can instantaneously recover its gripping performance once the gripper is unloaded.

There remain several future works to improve the proposed SSG with better performance. For example, as suggested by results in Figures 5 and 6, the use of a composite materials that consists of a nonstretchable backing (i.e., fabric or a thin layer of stiff elastomer) covered with a soft elastomer has a potential to further improve both suction force and adaptability on a broad range of rough surfaces. Also, the soft robotic architecture of the proposed SSG can easily be combined with various functional interfaces developed in previous works, such as electrostatically adhesive surfaces,^[^ ^6^, ^7^, ^45^ ^]^ crack trapping surfaces,^[^ ^46^, ^47^ ^]^ and microsuction surfaces,^[^ ^48^, ^49^ ^]^ to produce better performance in achieving robust and strong suction. Furthermore, characterization in gripping performance under various dynamic loading conditions will be an interesting topic, as a fast pulling speed can either reduce or increase the gripper's payload capacity by inertia of the object or viscoelasticity of the gripper body.^[^ ^15^ ^]^ We envision that the simplicity as well as the versatility of the proposed soft suction gripper in this research will serve as a cornerstone in developing universal soft robotic gripping devices that can provide an important functionality for the next generation of interactive robotic systems in the future.

## 4 Experimental Section

### Fabrication of the Soft Suction Gripper

The soft gripper body was obtained by replicating a negative mold (Figure S6, Supporting Information) as previously reported.^[^ ^40^ ^]^ In short, a negative mold made out of Ecoflex 00‐30 (Smooth‐On Inc.) was obtained by replicating a 3D‐printed composite model of the gripper body (GB). The gripper model was designed with a CAD software and created by a 3D printer (Objet260 Connex, Stratasys Ltd.) using a stiff (VeroClear) soft (TangoBlack) material.

The chamber was attached to a small plastic petri dish and a 1:1 ratio of Ecoflex 00‐30 prepolymer and crosslinker was mixed, degassed, and casted into the petri dish. After curing at room temperature for 6 h (Figure S6, Supporting Information) the gripper model was carefully demolded. The negative mold was treated in an oxygen plasma for 2 min, fluorosilanized for 1 h and cured at 90 °C for 30 min. The fluorosilanization of the mold allows the subsequent replication process. A thin metal bar was applied to the negative mold (Figure S6, v, Supporting Information) and a 1:1 ratio of Ecoflex 00‐50 (Smooth‐On Inc.) parts A and B were mixed, degassed and injected into of the negative mold with a syringe. After curing at room temperature for 14 h, the soft GB was demolded.

The SSG membranes were fabricated of materials with different Young´s moduli (Figure S7A, Supporting Information). A 1:1 ratio of Ecoflex 00‐50 (Smooth‐On Inc.) Parts A and B were mixed, degassed, and casted on perfluorinated silicon wafer. A 200 µm thick thin layer was created by a bar coater (K‐Hand‐Coater, Erichsen GmbH & Co. KG) and cured at room temperature for 14 h and peeled off. The Ecoflex 00‐10 and Sylgard 184 (Dow Chemical Co., Ltd.) membranes were fabricated as described before, but cured at room temperature for 6 h and cured in a vacuum oven at 90 °C for 1 h, respectively.

The membrane was bonded to the gripper body with a vinylsiloxane polymer (Flexitime Medium Flow, Heraeus Kulzer GmbH) (Figure S7B, Supporting Information). The vinylsiloxane precursor was applied on a glass plate and a thin film of 50 µm thickness was created by a film applicator (Multicator 411, Erichsen GmbH & Co. KG). The soft GB was manually inked into the polymer film and placed on a flat membrane. The vinylsiloxane covalently bonds the GB to the membrane after 5 min of curing at room temperature. The vinylsiloxane polymer was also used in assembling the connecting tube with the gripper body.

### Fabrication of the Rough Surface Replicas

A vinylsiloxane polymer (Flexitime medium flow, Heraeus Kulzer GmbH) was applied on a glass slide and an ≈2 mm thick film was created (Figure S4, Supporting Information). 1 mm thick spacers were attached on the edges of the glass slide. The vinylsiloxane film on top of the glass slide was pressed against a real surface, e.g., concrete, steel, ceramic, and polymer and cured for 3 min at room temperature (Figure S4‐ii, Supporting Information). After curing the sample was peeled off the surface. A 10:3 ratio of EpoxAcast 690 (Smooth‐On Inc.) prepolymer and crosslinker was mixed, degassed, and casted onto the surface replica, followed by another degassing step to ensure optimal replication quality. A glass slide with 1 mm thick spacers on the edges was placed on top, serving as a backing layer and cured at room temperature for 48 h (Figure S4‐v, Supporting Information). After demolding a positive replica of the rough surfaces was obtained.

### Experimental Setup

The soft gripper was characterized with a customized adhesion setup described before.^[^ ^40^ ^]^ In short, the adhesion setup was mounted on an inverted optical microscope (Axio Observer A1, Zeiss) with a video camera (Grasshopper3, Point Grey Research Inc.). The adhesion was measured by high‐resolution load cells (GSO‐25, GSO‐500, and GSO‐1K, Transducer Techniques), which was attached on a computer‐controlled high‐precision piezo motion stage (LPS‐65 2”, Physik Instrumente GmbH & Co. KG) in z‐direction. The substrate was placed on a sample holder and moved in *x*‐direction by the piezo stage (LPS‐65 2”, Physik Instrumente GmbH & Co. KG). Fine positions in *x*‐ and *y*‐direction and angular alignment were performed by a manual *xy*‐stage (NFP‐2462CC, Positionierungstechnik Dr. Meierling) and by two goniometers (M‐GON65‐U, Newport), respectively. The pressure inside the SSG was controlled by a syringe pump (Legato 210P, KDScientific Inc.) and measured with a pressure sensor (HSCSANN600MDAA5, Honeywell International Inc.). The same syringe pump is also used for pick‐and‐place manipulation in Movies S1 and S2 (Supporting Information). The motion of the piezo stages and the data acquisition were performed by a customized code in Linux (Ubuntu, Canonical Ltd.).

### Finite Element Method (FEM) Simulations

The simulation studies were conducted in a commercial FEM software (COMSOL Multiphysics, COMSOL Inc.). In the simulation, the geometry of the substrate (Figure 6A‐I) to yield a targeted RMS roughness value, *R*_q_, a sinusoidal surface shape was used. While the wavelength of the sinusoidal wave is given by the specific cut‐off wavelength (*λ*_c_), the amplitude of the wave (*R*_z_) is calculated to match the targeted *R*_q_ value. Ten waves on the substrate surface were used to test the conformability of the membrane on the substrate. Also, *λ*_c_ is assumed to be substantially smaller compared to the size of the flat membrane. With this assumption, the effect of the size of the membrane on the conformability is negligible. In the simulation, PDMS was used as a rigid membrane, while Ecoflex 00‐30 was used as a soft membrane, considering similar mechanical properties to both Ecoflex 00‐10 and Ecoflex 00‐50, and its Young's modulus lies in the middle of the Young's moduli of the two elastomers.^[^ ^39^, ^50^ ^]^ To simulate the nonlinear material behaviors of PDMS and Ecoflex 00‐30, the 5‐parameter Mooney‐Rivlin hyperelastic model is employed. The pressure applied to the top part of the membrane induces the deformation of the membrane. The pressure is increased iteratively until the gap between the membrane and the substrate closes. The gap is determined to be closed when the maximum distance between the membrane and the substrates goes below 9 nm. During the whole simulation, the boundary condition at both ends was kept frictionless, using roller constraints in COMSOL. The linear material simulation (Figure 6E) is conducted in a same way as described above, except the materials behavior is assumed to be linear. For the numerical stability in the simulation, still the Mooney‐Rivlin hyperelastic model is employed, while the stress and strain relationship kept linear by fitting Mooney‐Rivlin coefficients to the linear line with a slope of a given Young's modulus.

### Statistical Analysis

Continuous variables are expressed as mean ±1 standard deviation (SD) for five measurements (*n* = 5). *t‐*tests are carried out in comparison of two normally distributed data sets with equal variances. In all cases, significance was defined as *p* ≤ 0.05. Statistical analysis is carried out using the Origin Software.

## Conflict of Interest

The authors declare no conflict of interest.

## Author Contributions

S.S., D.S., and D.‐M.D. contributed equally to this work. S.S., D.‐M.D., and M.S. proposed and designed the research; S.S., D.‐M.D., D.S., and A.K. performed the experiments; S.S. and D.‐M.D. analyzed the experimental data; D.S. performed the numerical analysis; S.S., D.‐M.D., D.S., and M.S. wrote the paper.

## Supporting information

Supporting Information

Supplemental Movie 1

Supplemental Movie 2

Supplemental Movie 3

Supplemental Movie 4

Supplemental Movie 5

## Data Availability

The data that supports the findings of this study are available in the supplementary material of this article.
