# Supplementary material for: Adaptive Self‐Sealing Suction‐Based Soft Robotic Gripper
Source: Adv Sci (Weinh). 2021 Jul 3;8(17):2100641. doi: 10.1002/advs.202100641 (PMC8425915; doi:10.1002/advs.202100641)
Supplement: Supplementary file 1 — Supporting Information [file ADVS-8-2100641-s005.pdf]

© 2021 Wiley-VCH GmbH

ADVANCED SCIENCE
Open Access

Supporting Information

for *Adv. Sci.*, DOI: 10.1002/advs.202100641

Adaptive Self-Sealing Suction-Based Soft Robotic Gripper

*Sukho Song, Dirk-Michael Drotlef, Donghoon Son, Anastasia Koivikko, and Metin Sitti**

Supporting Information

Adaptive Self-sealing Suction-based Soft Robotic Gripper

Sukho Song, Dirk-Michael Drotlef, Donghoon Son, Anastasia Koivikko, and Metin Sitti

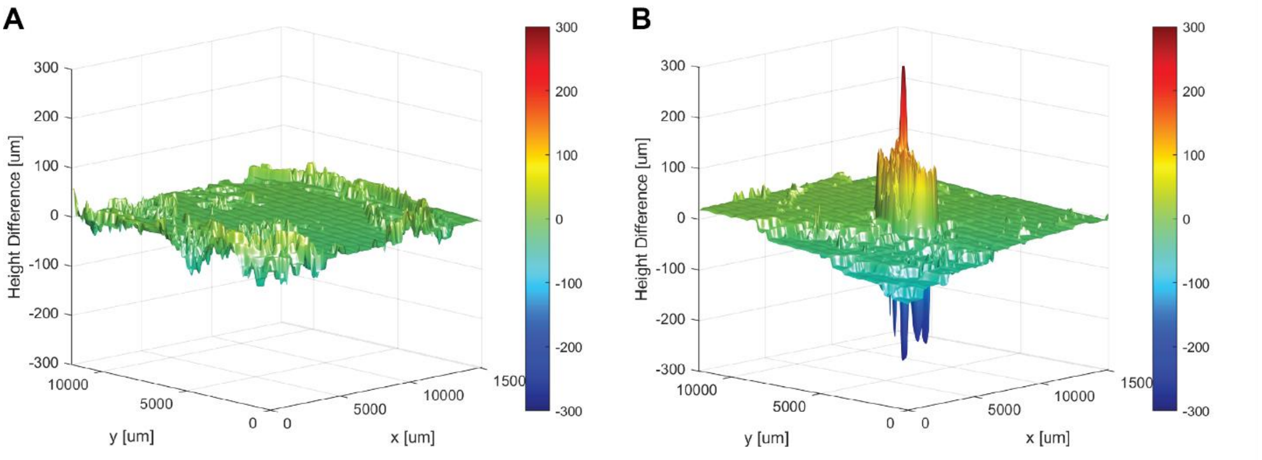

Figure S1. A comparison of damage on hydrogel surface depending on suction cup design. (**A**) A 3D topology of gelatin surface (5 wt%) after contacting with the soft suction gripper. (**B**) A 3D topology of gelatin surface (5 wt%) after contacting with the gripper body without the flat membrane.

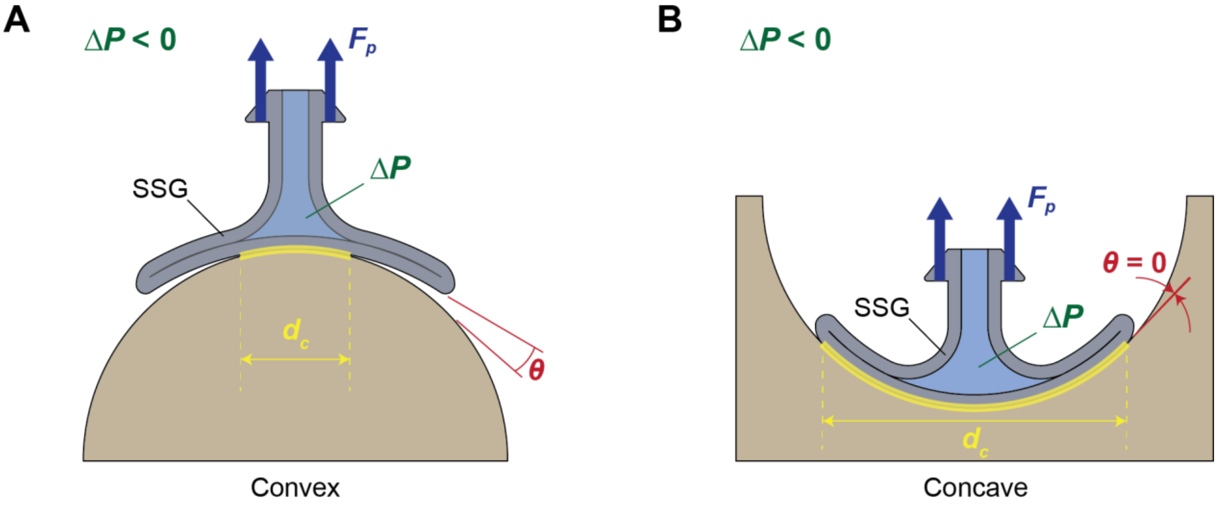

Figure S2. Schematics of the soft suction gripper (SSG) engaging two different types of 3D surface curvatures. (A) The SSG under a negative pressure differential ($\Delta P < 0$) grasping a convex surface at a pull-off force (F_p). The SSG cannot make a full contact with the curved surface, which ends up the diameter of contact interface (d_c) smaller than the gripper size. Also, the peeling angle (θ) increases at the edge of the gripper, resulting in a reduced pull-off force. (B) The SSG under a negative pressure differential ($\Delta P < 0$) grasping a concave surface at a pull-off force (F_p). The SSG makes a full contact to the curved surface with the diameter of contact interface (d_c) equal to the gripper size. Also, the peeling angle (θ) remains zero ($\theta = 0$) over the entire contact interface, which leads to a higher pull-off force (F_p) than that of the SSG on a convex surface.

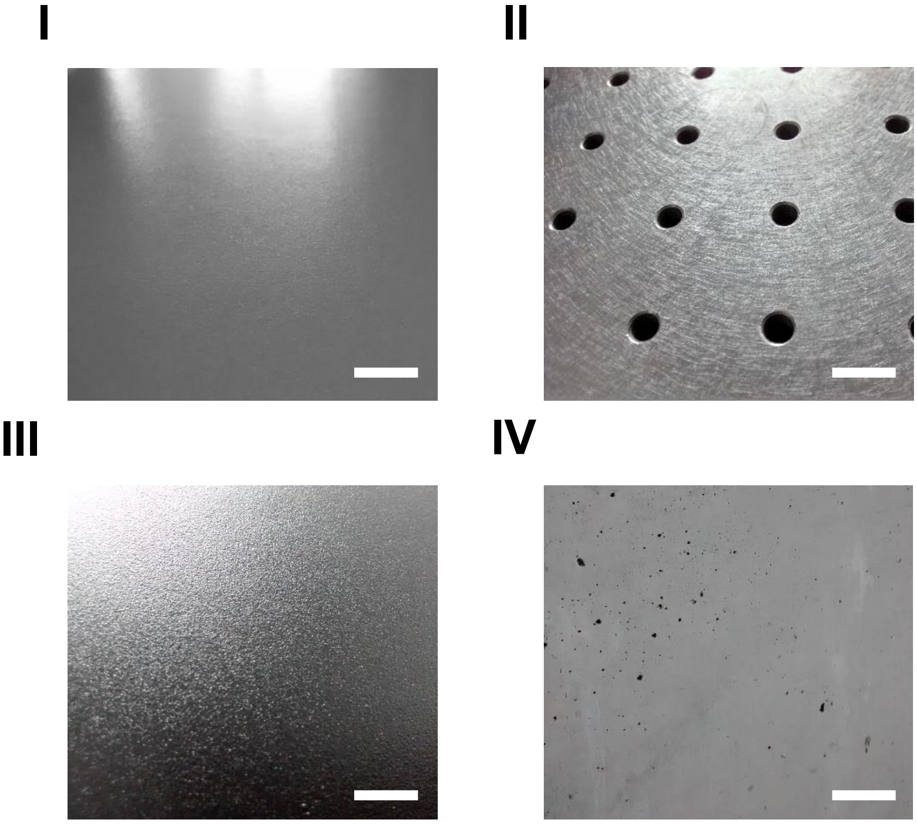

Figure S3. Top-view photographs of the rough surfaces used in the attachment tests. (**I**): RS 1 ceramic, (**II**): RS 2 steel, (**III**): RS 3 polymer, and (**IV**): RS 4 concrete. Scales indicate 10 mm.

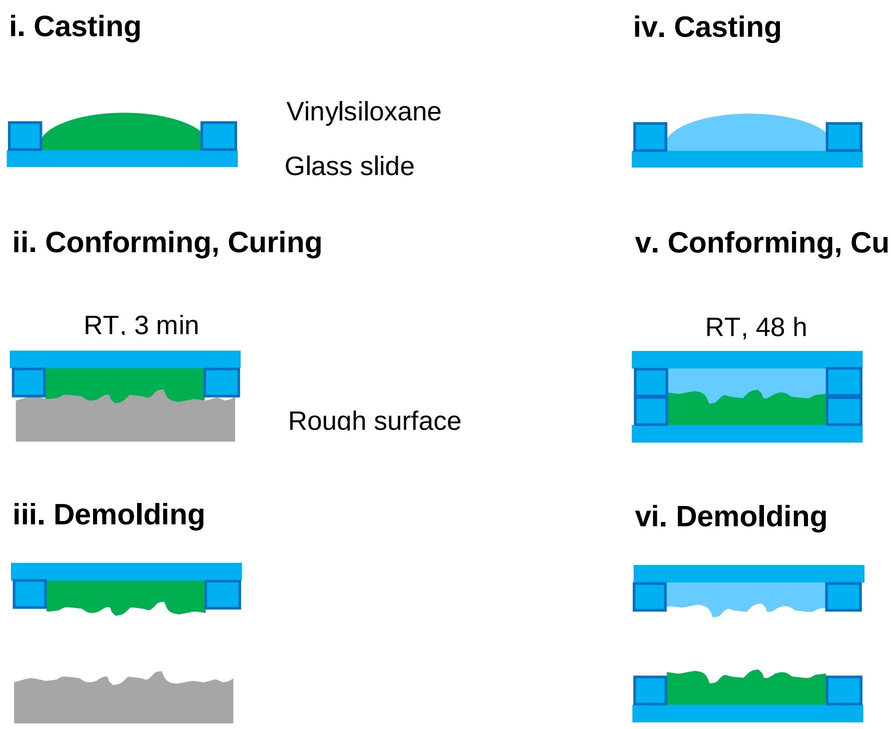

Figure S4. Fabrication process of the rough surface replicas. Schematic demonstrates the individual fabrication steps. Fabrication of the vinylsiloxane negative mold (steps i–iii), molding and demolding of the positive rough surface replica made of the epoxy polymer (EpoxAcast^{TM} 690, Smooth-on Inc.) (steps iv-vi).

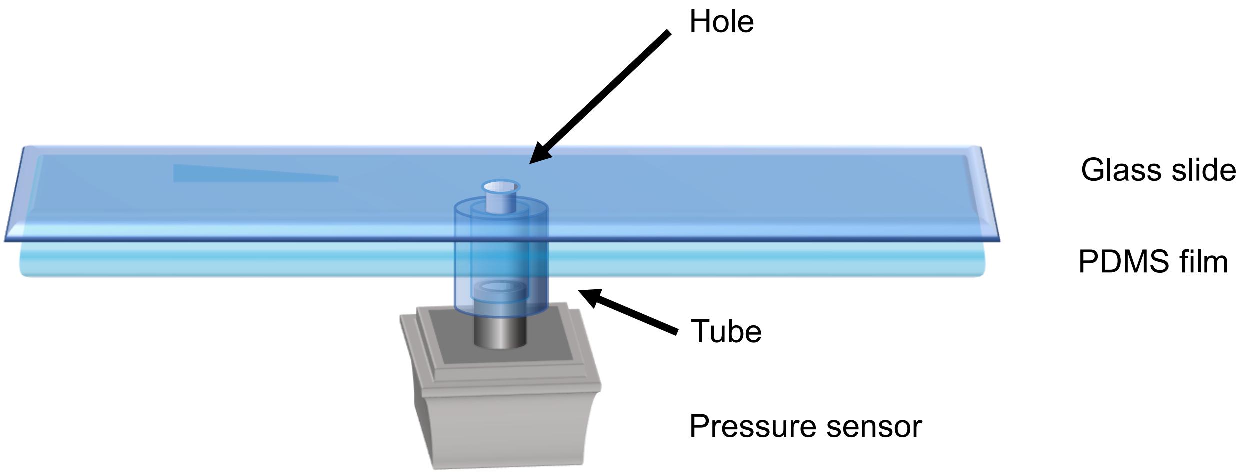

Figure S5. Schematic of the setup for measuring the interfacial pressure. A pressure sensor is located below the 500 µm diameter hole in the glass slide and attached via a silicon tube. The tube is embedded in a PDMS film, which ensures proper attachment to the glass slide.

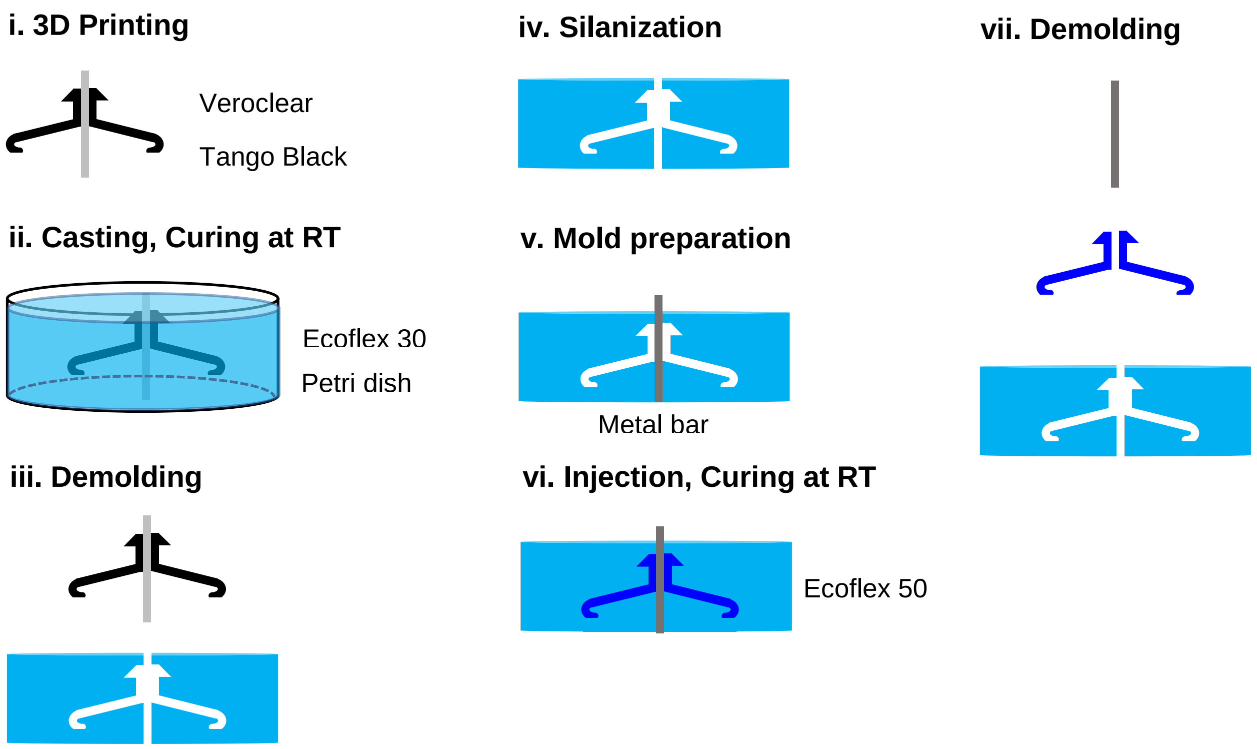

Figure S6. Fabrication process of the soft gripper body. Schematic demonstrates the individual fabrication steps. Fabrication of the Ecoflex 30 negative mold (steps i–iv), molding and demolding of the positive gripper body made of Ecoflex 50 (steps v-vii).

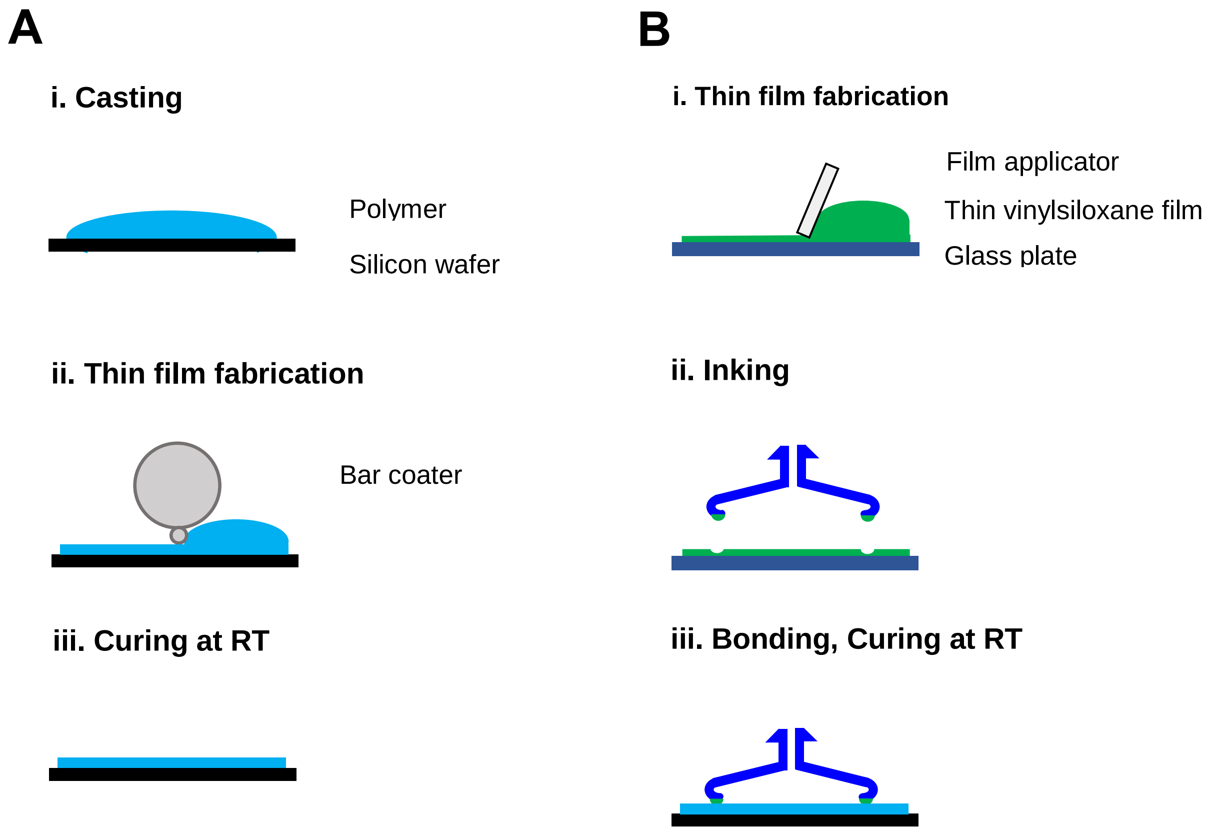

Figure S7. Fabrication process of the gripper membrane and the gripper assembly. Schematic shows the individual fabrication steps. Fabrication of the membranes **A** (steps i–iii) and bonding of the gripper to the membrane **B** (steps i-iii).

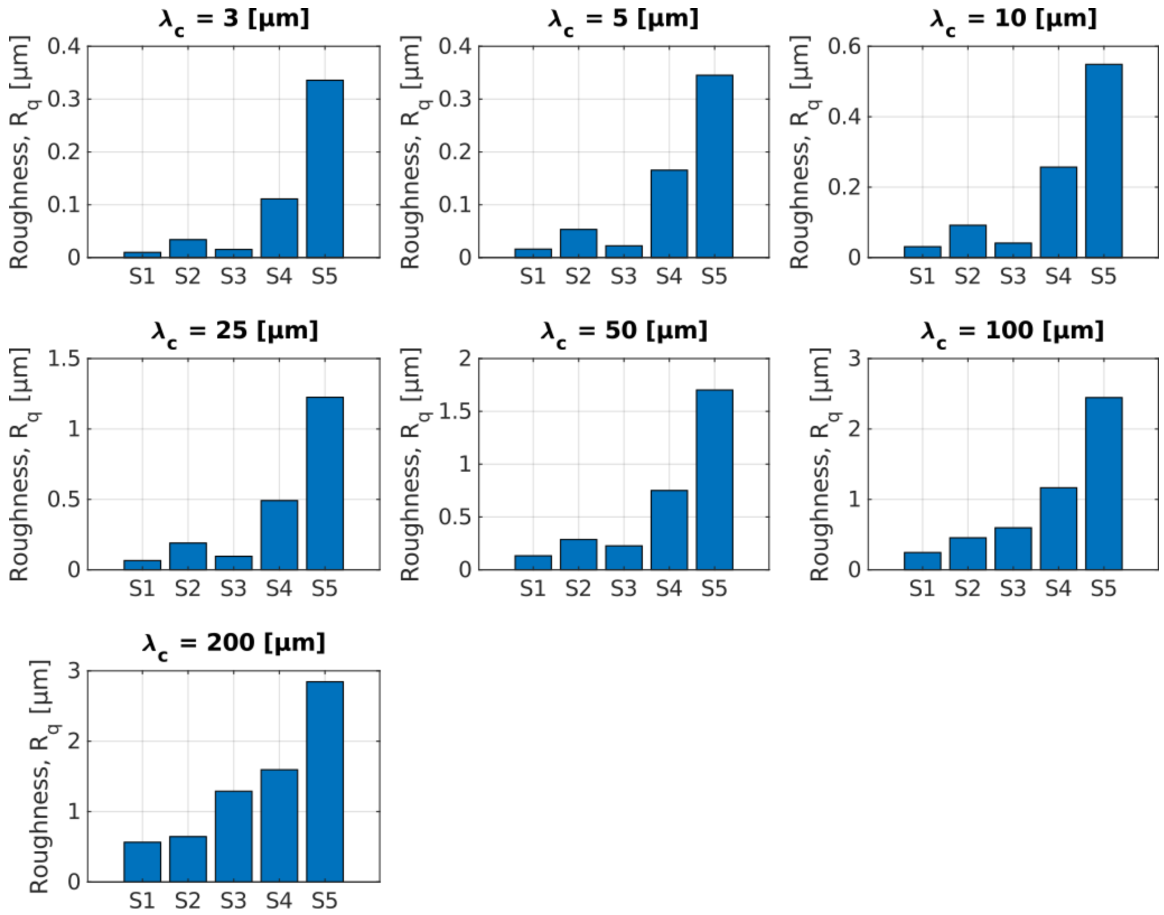

Figure S8. Experimentally measured RMS roughness values of the tested five different roughness replicas (RS1-RS5) depending on different cut-off wavelengths (λ_c). The trend from microscopic (3 μm) to macroscopic (200 μm) RMS roughness values shows that the tested rough surfaces are composed of various length-scale micro- and macroscopic roughness values.

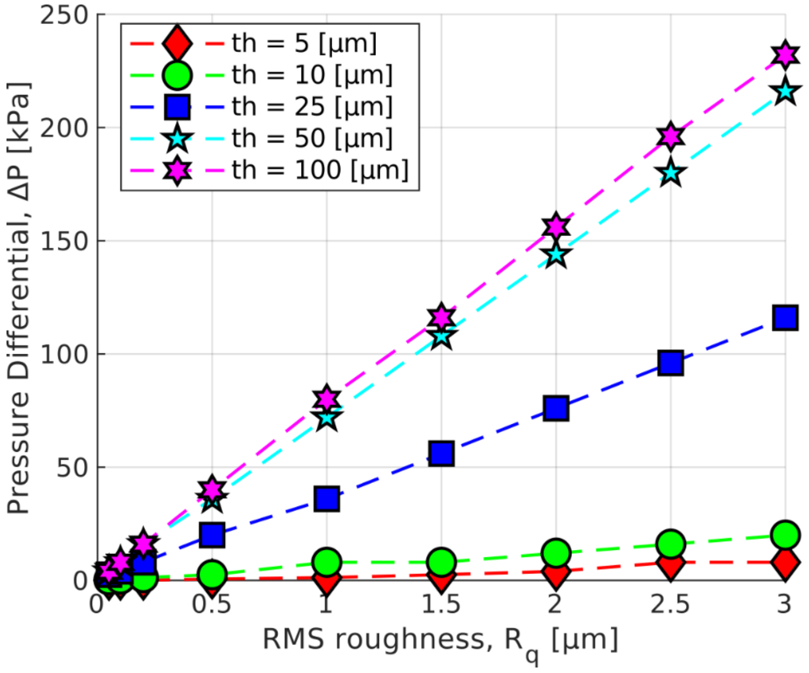

Figure S9. Conformability of the PDMS membrane on a rough surface with the macroscopic rough structures (λ_c = 100 μm) as a function of the membrane thickness (5-100 μm). The experimental results suggest that the surface conformability is enhanced by reducing the thickness of the PDMS membrane. This allows the SSG with the PDMS membrane thinner than 10 μm to grip RS 3.

Table S1. Pull-off force (F_p) of the SSG with and without the membrane on various surfaces (rough surface (RS) 1-5 and smooth glass (SG)) (**Figure 4C**), and their p-values between the SSG and the Gripper Body Only. Each point is an average of five measurements (n=5) with corresponding standard deviation (SD). P-values are calculated by *t*-test using Origin software.

Surface Type	SSG		Gripper Body Only		*p*-Value
	F_p	SD	F_p	SD	
SG	2.75	0.17	0.71	0.01	3.88×10^{-9}
RS1	2.69	0.09	0.72	0.16	8.25×10^{-9}
RS2	2.61	0.07	0.78	0.02	1.42×10^{-11}
RS3	2.32	0.25	0.73	0.03	5.57×10^{-7}
RS4	2.03	0.38	0.41	0.03	1.41×10^{-5}

Supplementary Movies

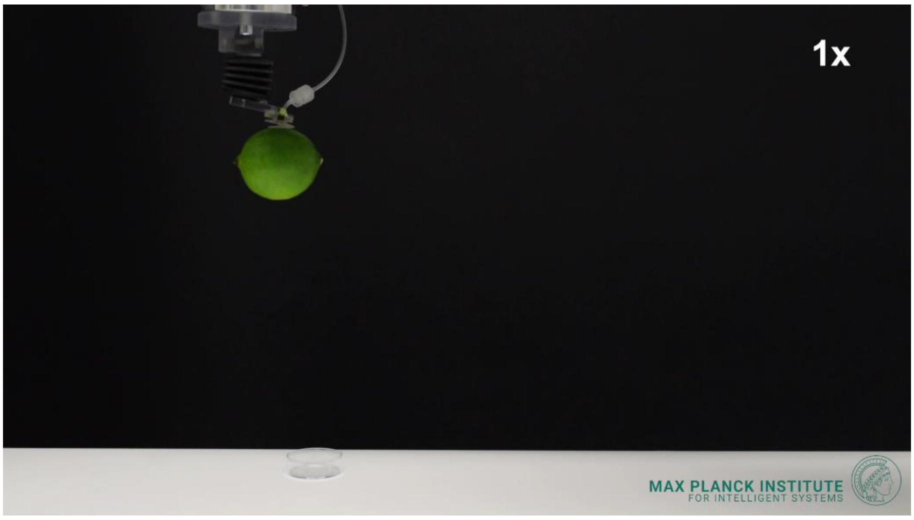

Movie S1. The proposed soft suction gripper mounted on a robotic arm, manipulating a real-world 3D part with surface texture (53-gram lime).

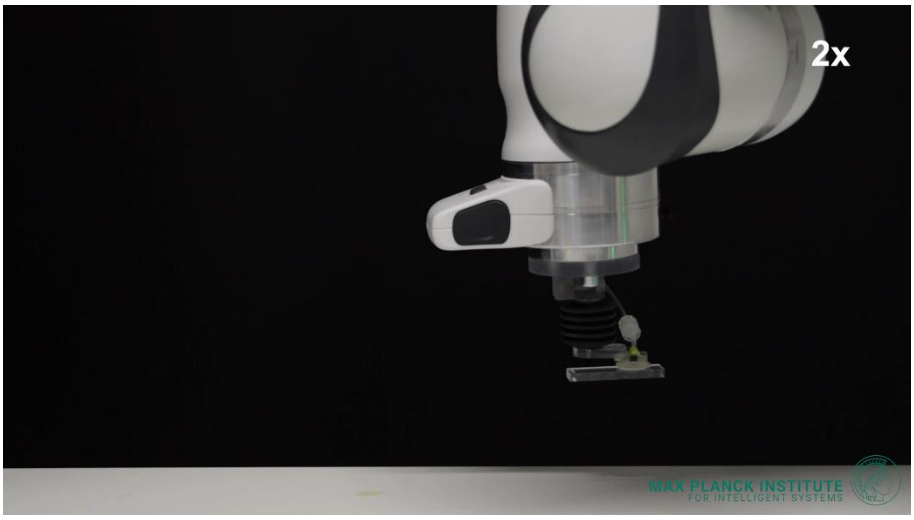

Movie S2. The soft suction gripper mounted on a robotic arm, manipulating parts smaller than the gripper diameter (two rectangular-shaped PMMA bars, 14.3 mm and 5.0 mm in width, respectively).

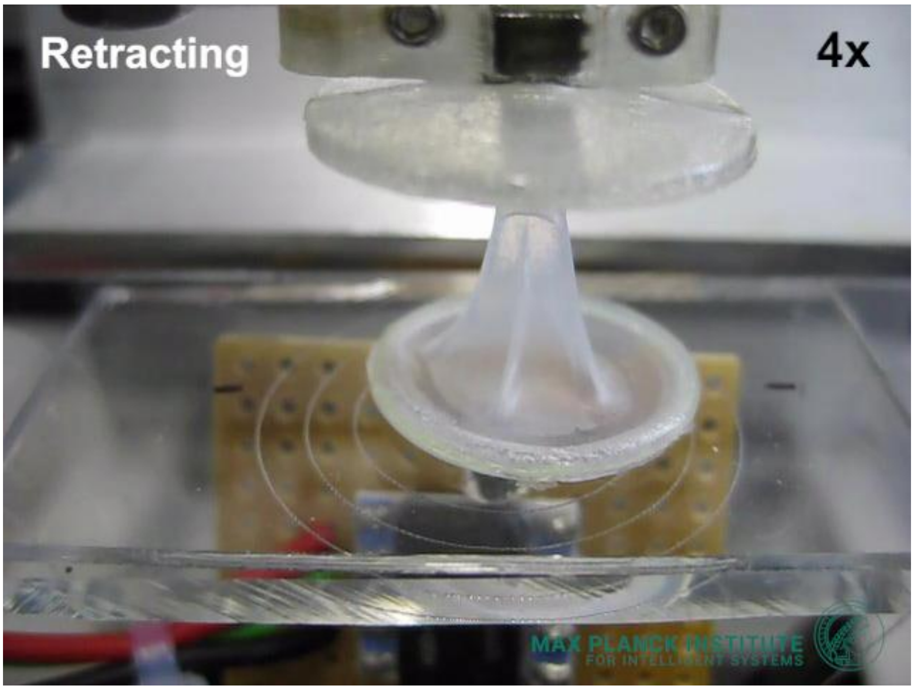

Movie S3. An experimental pull-off force measurement of the soft suction gripper on a flat smooth glass surface.

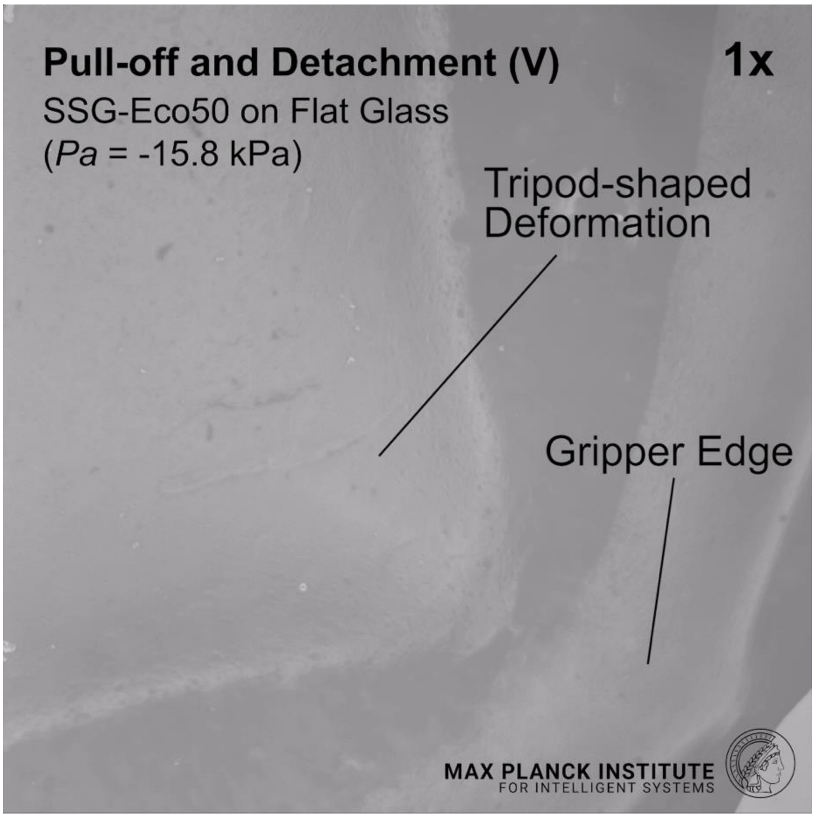

Movie S4. A microscopic view of the soft suction gripper at the tip of a tripod-shaped deformation nearby the gripper edge during detachment. Under a high pulling load, a locally

concentrated stress creates a small breach that eventually propagates over the entire contact interface.

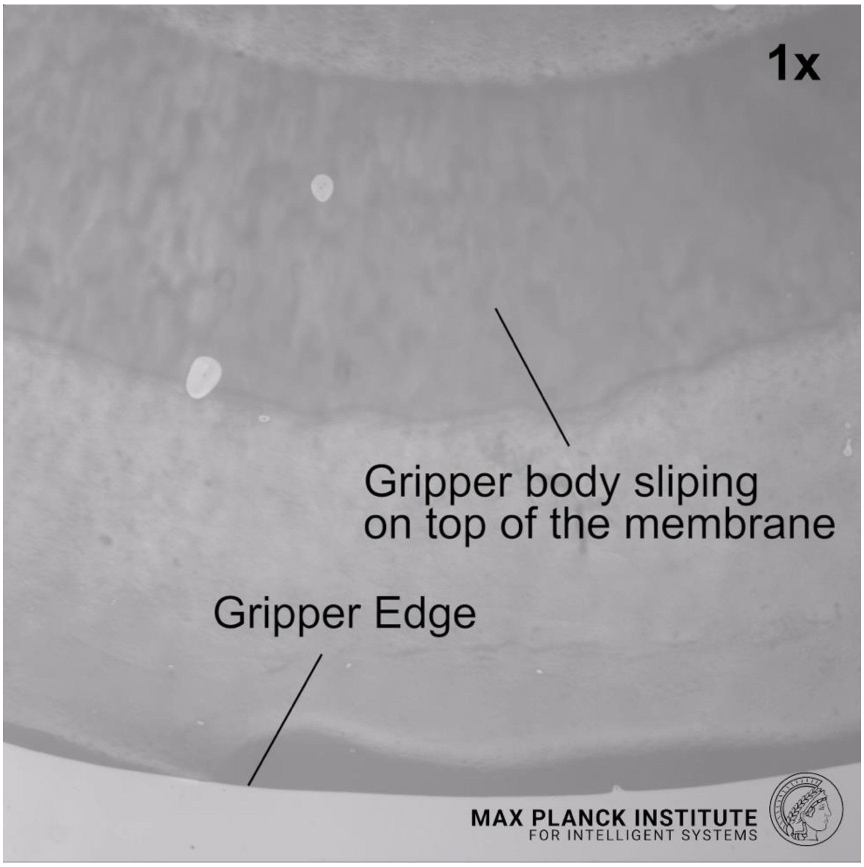

Movie S5. A microscopic view of the soft suction gripper near by the gripper edge during detachment, showing random stick-and-slip of the soft gripper body under a high pulling load. A dark area represents the gripper body in contact with the flat membrane of the soft gripper, while a bright area shows the gripper body not contacting the membrane.
